# Bioinformatic Analyses of miRNA–mRNA Signature during hiPSC Differentiation towards Insulin-Producing Cells upon HNF4α Mutation

**DOI:** 10.3390/biomedicines8070179

**Published:** 2020-06-27

**Authors:** Luiza Ghila, Yngvild Bjørlykke, Thomas Aga Legøy, Heidrun Vethe, Kenichiro Furuyama, Simona Chera, Helge Ræder

**Affiliations:** 1 Department of Clinical Science, Faculty of Medicine, University of Bergen, 5021 Bergen, Norway; Yngvild.Bjorlykke@uib.no (Y.B.); Thomas.Legoy@uib.no (T.A.L.); Heidrun.Vethe@uib.no (H.V.); simona.chera@uib.no (S.C.); 2 Department of Genetic Medicine & Development, Faculty of Medicine, University of Geneva, 1211 Geneva, Switzerland; kenichir@kuhp.kyoto-u.ac.jp; 3 Department of Pediatrics, Haukeland University Hospital, 5021 Bergen, Norway

**Keywords:** miRNA, hiPSC, HNF4α, MODY1, insulin-producing cells, in-vitro differentiation, endocrine, progenitor, pancreas, pluripotent stem cells

## Abstract

Mutations in the hepatocyte nuclear factor 4α (HNF4α) gene affect prenatal and postnatal pancreas development, being characterized by insulin-producing β-cell dysfunction. Little is known about the cellular and molecular mechanisms leading to β-cell failure as result of HNF4α mutation. In this study, we compared the miRNA profile of differentiating human induced pluripotent stem cells (hiPSC) derived from HNF4α^+/Δ^ mutation carriers and their family control along the differentiation timeline. Moreover, we associated this regulation with the corresponding transcriptome profile to isolate transcript–miRNA partners deregulated in the mutated cells. This study uncovered a steep difference in the miRNA regulation pattern occurring during the posterior foregut to pancreatic endoderm transition, defining early and late differentiation regulatory windows. The pathway analysis of the miRNAome–transcriptome interactions revealed a likely gradual involvement of HNF4α^+/Δ^ mutation in p53-mediated cell cycle arrest, with consequences for the proliferation potential, survival and cell fate acquisition of the differentiating cells. The present study is based on bioinformatics approaches and we expect that, pending further experimental validation, certain miRNAs deregulated in the HNF4α^+/Δ^ cells would prove useful for therapy.

## 1. Introduction

Numerous studies aimed at deciphering the molecular regulation of various biological processes have proven that, besides protein–protein interactions, microRNAs (miRNAs) are also essential for regulating cellular molecular machinery. miRNAs are 19–22-nucleotide noncoding RNAs that regulate gene expression post-transcriptionally by base pairing with complementary sequences in the 3′ untranslated regions (3′UTRs) of protein-coding transcripts, thereby usually inhibiting their translation and/or stability [1]. Some of these noncoding RNA molecules have already been shown to play major roles in the regulation of β-cell development [2,3,4], maintenance and function [5,6,7], including insulin secretion [8] and circadian clocks [9]. The significance of miRNAs during in-vitro differentiation to insulin-expressing cells was previously illustrated by experiments in human pluripotent stem cells, which showed that miR-200a has a critical role in regulating both epithelial–mesenchymal transition and definitive endoderm formation through direct repression of ZEB2 and SOX17, while miR30d and let-7e regulate the pancreatic progenitor gene RFX6 during late-stage differentiation [10]. Other studies showed that healthy hiPSCs [11] differentiating into insulin-producing cells presented a specific signature during this process, and the differentially expressed miRNA target genes were involved in endocrine pancreatic organogenesis. For example, miRNA-690 was shown to regulate induced pluripotent stem cells’ (iPSCs) differentiation into insulin-producing cells by targeting Sox9, previously linked to proliferation and differentiation of endocrine progenitors [12]. Moreover, the miR-302 cluster, previously involved in pluripotent stem cell maintenance and in the acquisition of undifferentiated phenotype, was also shown to have a role in the in-vitro dedifferentiation of human pancreatic islet cells [13]. All these studies indicate that a certain cell fate choice can be defined by a particular miRNA signature [14]. However, specific alterations of the miRNA cellular landscape in different forms of diabetes are yet to be investigated.

Mutations in the hepatocyte nuclear factor 4A (HNF4α) gene lead to maturity-onset diabetes of the young (MODY)-1, characterized by pancreatic beta cell dysfunction affecting both prenatal and postnatal pancreas development [15]. Despite the comprehensive characterization of the mutation sites leading to the diabetic condition, very little is known about the cellular and molecular mechanisms responsible for the disease’s onset and progression. The main limitations are the ethical concerns regarding research on patients as well as the lack of suitable animal models [16,17,18,19]. Consequently, efforts are being made to employ human induced pluripotent stem cells (hiPSCs) as a model system for diabetes development. This is achieved by guiding human hiPS-cells derived from MODY1 families (i.e, mutation carrier patients and healthy control siblings) through fetal pancreas development in a stepwise fashion [20]. It is expected, but not yet demonstrated, that mature β-cells derived from MODY1 patients’ hiPSCs should mirror the reduced insulin secretion observed in MODY1 patients. Correspondingly, comparative analysis of MODY1-iPSC-derived pancreatic cell stages and lines may reveal the underlying, disease-specific mechanisms and pathways. We previously showed that insulin-positive cells could be generated in vitro from hiPSCs derived from patients carrying a nonsense HNF4α mutation, proving that the heterozygous state of p.Ile271fs mutation in human HNF4α is neither blocking the expression of the insulin genes nor the in-vitro development of insulin-producing cells [21]. Another study also showed that loss of HNF4α-mediated gene regulation affected foregut endoderm gene expression signatures, impairing liver and pancreas cell differentiation [22]. Recently, HNF4α was involved in controlling cell fate selection of pancreatic progenitors in vivo, upon transplantation in live hosts, by confining the hormone expression choice [23]. 

Here, we present miRNA signatures of MODY1-hiPSC spanning seven stages of in-vitro differentiation along the pancreatic beta cell lineage (from definitive endoderm to hormone-expressing maturing endocrine islet cells) in samples derived from a MODY1 family (healthy control sibling (C) and two mutation carriers, HNF4α^+/Δ^). We show that the miRNA landscape in MODY1-generated differentiating cells is different from that in healthy hiPSC-derived hormone-producing islet cells and that their expression profile is drastically changed during the transition from the posterior gut to the pancreatic endoderm, defining two differentiation windows based on their regulation pattern. Moreover, by superimposing the corresponding transcriptome landscape, we performed the pathway analysis of these early and late differentiation windows and focused on transcript–miRNA couples deregulated in the mutated cells.

## 2. Experimental Section

### 2.1. Cell Source

The experiments performed in this study were approved by the Norwegian Regional Committee of Medical and Health Research Ethics (5 October 2010, REK 2010/2295) and were performed in accordance with the Helsinki Declaration, and informed consent was obtained from the patients and healthy donors. We used human induced pluripotent stem cells (hiPSCs) previously reprogrammed from skin fibroblasts donated by two HNF4α mutation carriers (male patients) and one family male sibling (healthy control) [21]. The characteristics of the donors are presented below, in Table 1.

The iPSCs cell lines were characterized and confirmed to have a normal karyotype and to be mycoplasma free using a MycoAlert Mycoplasm Detection Kit (Lonza, Basel, Switzerland, LT07-418). All hiPSC lines were assessed for pluripotency and enriched in SSEA4+ population (SSEA4 microbeads, 130097855, Miltenyi Biotec Norden, Lund, Sweden) before differentiation.

### 2.2. In-Vitro Differentiation

All three iPSC lines were differentiated in parallel on Matrigel-coated 6-well plates for the duration of the entire differentiation (28 days). iPSCs were maintained in mTeSR medium (STEMCELL Technologies, Vancouver, BC, Canada, cat. no. 85850) on a thin layer of Matrigel Basement Membrane Matrix Growth Factor Reduced (40 µg/cm^2^, Corning, NY, USA, cat. no. 356230).

At 48 h before differentiation, iPSC cultures of about 75% confluence were rinsed with 1× DPBS without Mg2+ and Ca2+ (Sigma, St. Louis, MO, USA, cat. no. D8537), followed by incubation with TrypLE Express Enzyme (1×) (Gibco, Carlsbad, CA, USA, cat. no. 12563-011) for 4–5 min at 37 °C. Single iPSCs were collected in mTeSR medium and spun down at 500g for 5 min. The resulting cell pellet was resuspended in mTeSR medium supplemented with 10 mM ROCK inhibitor Y-27632 (Sigma, St. Louis, MO, USA, cat. no. Y0503), and the single-cell suspension was seeded at ~1.5–2 × 10^6^ cells/well on Matrigel-coated 6-well plates. The mTeSR medium was changed once following seeding, resulting in about 90% confluence per well before differentiation started at 48 h post-seeding.

Days 1–3: Undifferentiated hiPSCs were cultivated in the differentiation medium, containing MCDB131 medium (Life Technologies, Carlsbad, CA, USA, cat. no. 10372-019), 1.5 g/L sodium bicarbonate (Sigma, St. Louis, MO, USA, cat. no. S6297), 1× Glutamax (Life Technologies, cat. no. 35050-079), 10mM glucose (Sigma, St. Louis, Missouri, USA, cat. no. G7021) concentration and 0.5% BSA (Sigma, St. Louis, MO, USA, cat. no. A7030). For day 1 and day 2 the medium was supplemented with 100 ng/mL Activin A (PeproTech Nordic, Stockholm, Sweden, cat. no. 120-14) and 0.3µM CHIR-99021 (Selleckchem, Houston, TX, USA, cat. no. S2924). For day 3 only, 100 ng/mL Activin A was added to the differentiation medium. Samples for RNA extraction were collected at the end of day 3 (for stage 1: definitive endoderm) following incubation with TrypLE Express Enzyme (1×) for 5 min at 37 °C. Single iPSCs were collected in PBS, spun down at 250× *g* for 5 min, resuspended in 70 µL of lysis buffer (Qiagen, Hilden, Germany, miRNeasy cat. no. 217084) and stored at −80 °C until use for RNA extraction.

Days 4–5: For the differentiation towards primitive gut tube, the cells were cultivated in the differentiation MCDB131 medium supplemented with 0.25 mM Ascorbic acid (Sigma, St. Louis, MO, USA, cat. no. A4544) and 50ng/mL FGF7 (PeproTech Nordic, Stockholm, Sweden, cat. no. 100-19) for 2 days. The medium was changed once. At the end of day 5, samples were collected and stored as described above.

Days 6–7: Cells were further cultivated in the differentiation MCDB131 medium with 2.5 g/L sodium bicarbonate, 1× Glutamax, 10 mM glucose, 2% BSA, 1 mM retinoic acid (RA; Sigma, St. Louis, MO, USA, cat. no. R2625), 0.25 mM ascorbic acid, 50 ng/mL of FGF7, 0.25 mM SANT-1 (Sigma, St. Louis, MO, USA, cat. no. S4572), 100nM LDN193189 (BMP receptor inhibitor, Stemgent, Cambridge, MA, USA, cat. no. 24804-0079), 1:200 ITS-X (Life Technologies, Carlsbad, CA, USA, cat. no. 51500056), and 200 nM TPB (PKC activator; Tocris, Bristol, United Kingdom, cat. no. 5343). The medium was changed every day. Samples of posterior foregut were collected at the end of day 7 and processed as described above.

Days 8–10: Cells were cultivated in the above medium with the following changes: 2 ng/mL of FGF7, 0.1mM retinoic acid, 200 nM LDN193189, and 100 nM TPB. Samples of pancreatic endoderm were collected at the end of day 10.

Days 11–13: S4 cells were cultivated further in MCDB131 medium with 1.5g/L sodium bicarbonate, 1× Glutamax, 20 mM glucose, 2% BSA, 0.25 mM SANT-1, 0.05 mM retinoic acid, 100nM LDN193189, 1:200 ITS-X, 1mM 3,3′,5-Triiodo-l-thyronine sodium salt (T3, Sigma, St. Louis, MO, USA, cat. no. T6397), 10 mM ALK5 inhibitor II (Enzo Life Sciences, Farmingdale, NY, USA, cat. no. ALX-270-445), 10mM zinc sulfate (Sigma, St. Louis, MO, USA, cat. no. Z0251) and 10 mg/mL of heparin (Sigma, St. Louis, MO, USA, cat. no. H3149). At the end of day 13, samples of pancreatic endocrine precursors were collected as described above.

Days 14–20: Cells were further differentiated in MCDB131 with 1.5 g/L sodium bicarbonate, 1× Glutamax, 20 mM glucose, 2% BSA, 1:200 ITS-X, 1mM T3, 100 nM LDN193189, 10mM ALK5 inhibitor II, 10mM zinc sulfate, 100 nM gamma secretase inhibitor (EMD Millipore/MERCK, Darmstadt, Germany, cat. no. 565789) and 10mg/mL of heparin. At the end of day 20, samples of immature hormone expressing islet-like cells were collected as described above.

Days 21–28: Cells were further differentiated in MCDB131 supplemented with 1.5 g/L sodium bicarbonate, 1× Glutamax, 20 mM final glucose concentration, 2% BSA, 1:200 ITS-X, 1 mM T3, 10 mM ALK5 inhibitor II, 10 mM zinc sulfate, 1 mM *N*-acetyl cysteine (N-Cys, Sigma, St. Louis, Missouri, USA, cat. no. A9165), 10 mM Trolox (Vitamin E analogue, EMD Millipore/MERCK, Darmstadt, Germany, cat. no. 648471), 2 mM R428 (AXL inhibitor, SelleckChem, Houston, TX, USA, cat. no. S2841) and 10 mg/mL of heparin. At the end of day 28, samples of maturing hormone-producing islet-like cells were collected as described above.

### 2.3. RNA Extraction

Samples representing one to six replicates of hiPSCs differentiating towards pancreatic islet-like cells (stages 1–7) were thawed on ice, and RNA was extracted using miRNeasy Micro (Qiagen, Hilden, Germany, cat. no. 217084) following the manufacturer’s instructions. Genomic DNA was removed using an RNase-free DNase Set (Qiagen, Hilden, Germany, cat. no. 79254). Total RNA concentration and integrity were checked using QIAxcel (Qiagen, Hilden, Germany,) and a 2100 Bioanalyzer (Agilent Technologies, Santa Clara, CA, USA).

### 2.4. miRNA Detection and Bioinformatics Analyses

Detection of miRNA was performed using the nCounter (NanoString, Seattle, WA, USA) at the Genomic Platform (University of Geneva). We analyzed RNA samples for miRNA using a human miRNA kit codeset NS_H_miR_v3a consisting of probes specific for 798 miRNAs, according to the manufacturer’s instructions (NanoString, Seattle, WA, USA). The reaction was carried out with 100 ng of the total RNA isolated as described above. The raw output .RCC files are available upon request. Pre-processing was performed as instructed by Nanostring for background correction. The Nanostring CodeSet contains negative controls, which are not specific to a target gene but contain alien sequences. The correction was done by subtracting the average signal plus 2 stdev of the negative controls from each miRNA in the dataset. The observed values less than 1 were given a threshold value of 1 in order to avoid negative values after log2 transformation. The log2 transformation was performed by importing the data in R bioconductor. The PCA and hierarchical clustering were performed using GeneSpring software (version 14.9.1 GX, Agilent Technologies, Santa Clara, CA, USA), with clustering on both entities and conditions, using the differential distance metric and Ward’s linkage rule. Differentially expressed miRNAs were identified using GeneSpring (fold change ≥ 1.5, *p* value ≤ 0.05), while TargetScan and microRNA.org were used to select target genes of differentially expressed miRNAs.

### 2.5. RNAseq and Bioinformatics Analyses

Total RNA samples were processed at the Genomic Platform (University of Geneva, Switzerland), using a HiSeq4000 machine (library UCSC-hg38), with 18 indexed libraries in two lanes using Illumina TruSec stranded mRNA (100 single-end reads) following the same procedure as described before [24,25]. Briefly, sequencing QC was done with FASTQC v.0.11.2. Reads were mapped with TopHat v2.0.13 default parameters to the reference genome on new junctions and known annotations. Biological QC and summarization were done with PicardTools v.1.80. Tables of counts were produced for aligned reads by Python software htseq-count v.0.0.6.1 with the reference gtf file (the multiple-mapping reads were not taken into account into the count table). The normalization and differential expression analysis were performed using the R-Bioconductor package EdgeR v.3.10.5 for the genes annotated in the reference genome. Hierarchical clustering was performed on both entities and conditions using GeneSpring GX version 14.9.1 GX (Agilent Technologies), using Ward’s method and differential metrics. The pathway analysis was performed using the Ingenuity Pathway Analysis program (IPA^®^, QIAGEN Redwood City, CA, USA) [26], as described in [25]. Briefly, we considered direct and indirect relationships, 35 molecules/network (25 networks), and all node types and data sources, and restricted the confidence to experimentally observed only.

### 2.6. Immunofluorescence Staining 

Differentiating cells were cultivated on Matrigel-covered glass coverslips (12 mm in diameter). At the end of each differentiation stage, the coverslips were washed three times with PBS (1×) and fixed with 4% paraformaldehyde for 15 min at room temperature. Then, cells were permeabilized by incubation with 1% Triton X-100 in 1× PBS for 15 min at room temperature. Cells were washed three times with 1× PBS and blocked with 2% BSA in 1× PBS for 1 h. Cells were incubated at 4 °C overnight with the following primary antibodies: goat anti-SOX17 (RD Systems, Minneapolis, MN, USA, cat. no. AF1924), mouse anti-GATA6 (RD Systems, Minneapolis, MN, USA, cat. no. MAB1700), goat anti-HNF1β (TCF-2) (RD Systems, Minneapolis, MN, USA, cat. no. AF3330), mouse anti-insulin IgG1 (1/500, cat. no. I2018, Sigma-Aldrich, St. Louis, Missouri, USA), guinea-pig anti-porcine insulin (1/400, cat. no. A056401-2, Dako, Glostrup, Denmark), mouse anti-porcine glucagon (1/1000, G2654, Sigma-Aldrich, St. Louis, Missouri, USA), goat anti-urocortin 3 (1/200, cat. no. ab79121, Abcam, Cambridge, United Kingdom), and rabbit anti-NKX6.1 (1/100, cat. no. NBP1-82553, Novus, St. Louis, MO, USA). Cells were washed three times with 1× PBS and incubated with a mix of DAPI (1/1000, cat. no. D1306, Molecular Probes, Eugene, OR, USA) and corresponding secondary antibodies (1/500) in a dark wet chamber at room temperature for 1 h. The following secondary antibodies were used: goat anti-mouse IgG1 A488, goat anti-guinea-pig A488, goat anti-mouse IgG1 A647, goat anti-guinea-pig A546, donkey anti-rabbit A647, and donkey anti-goat A647 (Molecular Probes, Eugene, OR, USA). Cells were washed three times with 1× PBS and mounted in Prolong Diamond Antifade Mountant Media (cat. no. P36970, Life Technologies, Carlsbad, CA, USA). Images were taken on a Leica TCS SP2 AOBS or Leica TCS SP5 STED CW confocal microscopes (Leica Microsystems, Wetzlar, Germany). No specific feature of the original data was obscured, eliminated or misrepresented.

### 2.7. Statistical Analyses

Unless otherwise specified, Mann–Whitney U tests were applied to graphed data using Prism 8.2.0 software (GraphPad Software Inc., La Jolla, California, USA). In figures, data are represented as mean ± SD.

## 3. Results

### 3.1. Stepwise In-Vitro Differentiation of HNF4α^+/Δ^-hiPSCs to Hormone-Producing Pancreatic Islet Cells 

**Figure 1:**
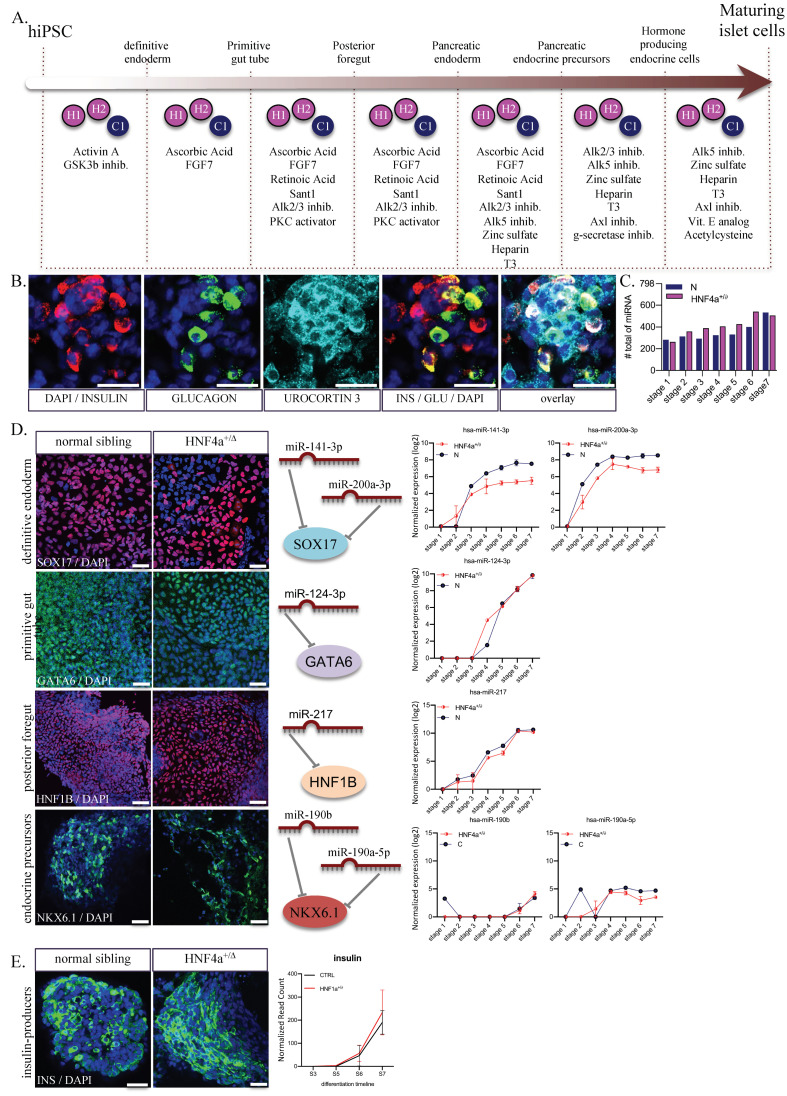
Differentiation of hiPSC towards pancreatic hormone-producing cells. (**A**) Step-wise differentiation scheme including the compounds used. (**B**) Immunostaining of maturing islet cells (stage 7) producing insulin (red); glucagon (green) and urocortin 3 (cyan). (**C**) Number of miRNAs expressed in the two conditions analyzed at each differentiation stage (*n* = 1–3 samples per stage for controls; *n* = 2–6 samples per stage for HNF4α^+/Δ^) (**D**) Immunostaining of SOX17 (definitive endoderm marker; red), GATA6 (foregut marker; green); HNF1B (pancreatic endoderm; red) and NKX6.1 (islet Β-cells). Middle panels depict the main miRNAs targeting the above-listed markers. Normalized read counts of selected miRNAs during differentiation in HNF4α^+/Δ^ or normal (blue) cells. Scale bars = 25 µm. (**E**) Immunostaining of insulin.

To address the mechanism of monogenic diabetes development, we previously established an in-vitro model based on derivation of several human induced pluripotent stem cell lines using fibroblasts from patients with MODY1 (maturity-onset diabetes of the young 1), carrying in a heterozygous state the p.Ile271fs mutation in *HNF4α* gene [20,21]. To monitor the adaptive variation of the miRNA signature, we used three different lines of MODY1-hiPSC generated from healthy (C) individuals and *HNF4α* mutation carriers (*HNF4α^+/Δ^*). We differentiated these lines towards hormone-producing maturing pancreatic islet cells (Figure 1A) by using a modification of a published seven-stage differentiation protocol [27,28]. Immunofluorescence staining confirmed expression of insulin, urocortin 3 and glucagon at the end of the differentiation protocol (day 28; Figure 1B). Samples were collected at each stage, and the miRNA expression was analyzed in 36 samples, including one to six replicates of hiPSCs differentiated toward pancreatic islet-like cells (stages 1–7). RNA was isolated from pelleted cells, and profiling for 798 miRNAs was performed using a Nanostring human miRNA panel (NS_H_miR_v3a). After quantile normalization, we identified between 263 and 541 miRNAs expressed in each of our conditions, with fewer miRNAs expressed at earlier stages of differentiation (Figure 1C). This could be explained by an increase in the complexity of the cell content, but also by increased heterogeneity of the cells undergoing differentiation in response to the added exogenous stimuli [21,29]. To briefly assess the miRNAs expressed during the differentiation, we selected several known markers for definitive endoderm (SOX17), posterior foregut (GATA6, HNF1B) and pancreatic endocrine precursors (NKX6.1) and searched TargetScan to identify their regulatory miRNAs (Figure 1D). The identified regulatory miRNAs (hsa-miR-141-3p and hsa-miR-200a-3p for SOX17; hsa-miR-124-3p for GATA6; hsa-miR-217 for HNF1B) showed, as expected, increased levels in later differentiation stages when targeted transcripts are expected to be downregulated (Figure 1D). The levels for hsa-miR-190b and hsa-miR-190a-5p (predicted to act on NKX6.1) are also raised and this might indicate a heterogeneous population in the later stages of differentiation. Of note, as previously reported by us [21], we observed no difference between healthy siblings and *HNF4α* mutation carriers regarding the production of insulin-positive cells, assessed here by immunostaining or by number of insulin transcripts (Figure 1E). 

### 3.2. The miRNAs’ Regulation Patterns Define Two Distinct Windows of Differentiation 

**Figure 2:**
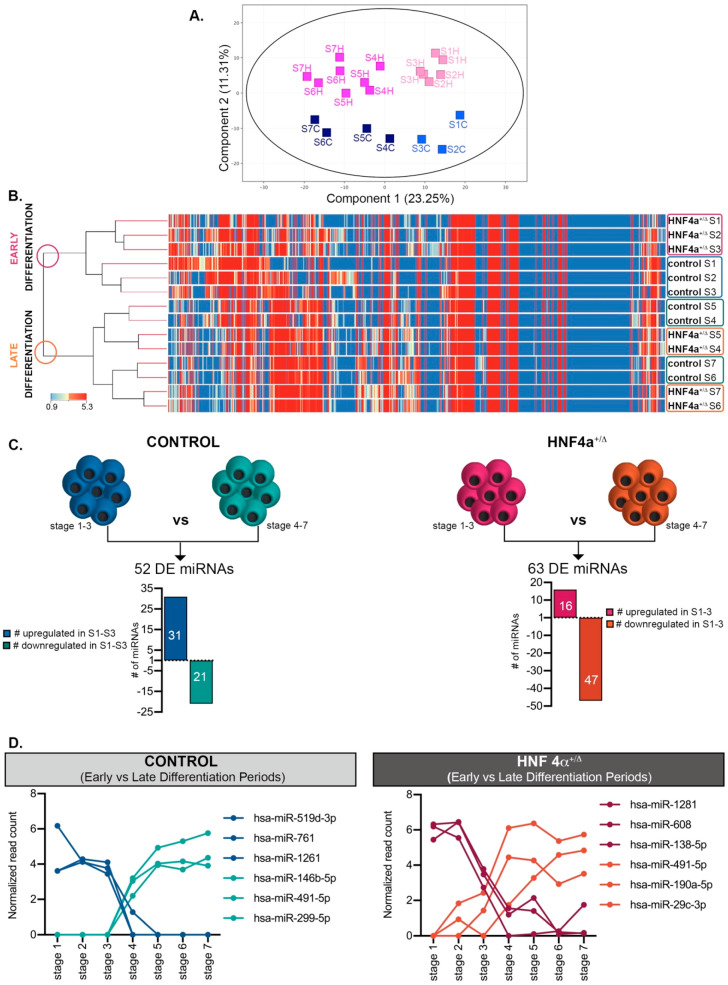
Analysis of the miRNAome during differentiation. (**A**) PCA plot of the conditions analyzed. (**B**) Hierarchical clustering of normalized miRNA expressed during the first seven stages of differentiation in HNF4α+/Δ and control-derived cells. (**C**) Number of miRNAs differentially expressed between early (S1–3) and late differentiation (S4–7) in either control- (left) or HNF4α^+/Δ^ −derived cells (right), *n* = 1–6 samples per stage. (**D**) Selected representative RNAs’ regulation patterns. Graphs’ data are given as mean ± SD.

To assess the samples variation, we generated a Principal Component Analysis (PCA) (Figure 2A), which identified a clear segregation of samples according to the differentiation interval (Principal Component 1: 23.25%, *X*-axis). Moreover, it revealed the separation of samples based on the HNF4α^+/Δ^ mutation status (Principal Component 2: 11.31%, *Y*-axis).

The hierarchical clustering reconfirmed these observations (Figure 2B), clustering the samples into two distinct main branches corresponding to early differentiation (stages 1 to 3) and late differentiation (stages 4 to 7) periods, with the mutation status being secondary to this separation. 

Based on these assays, we addressed the miRNA regulation pattern during differentiation. The analysis revealed that, indeed, many miRNAs changed their dynamic between the first (S1–3) and the last (S4–7) stages of differentiation, a period corresponding to the posterior foregut (stage 3) to pancreatic endoderm (stage 4) transition (Figure 2C,D). Interestingly, while in the healthy differentiating cells, more miRNAs were upregulated during the early stages of differentiation (31/52); the HNF4α^+/Δ^ cells exhibited opposing regulation, with most miRNAs (47/63) being upregulated during the late stages, indicating a strong involvement of the HNF4α^+/Δ^ mutation during the final stages of differentiation.

### 3.3. The HNF4α^+/Δ^ Mutation Status Modulates the miRNA Landscape in a Period-Dependent Manner 

**Figure 3:**
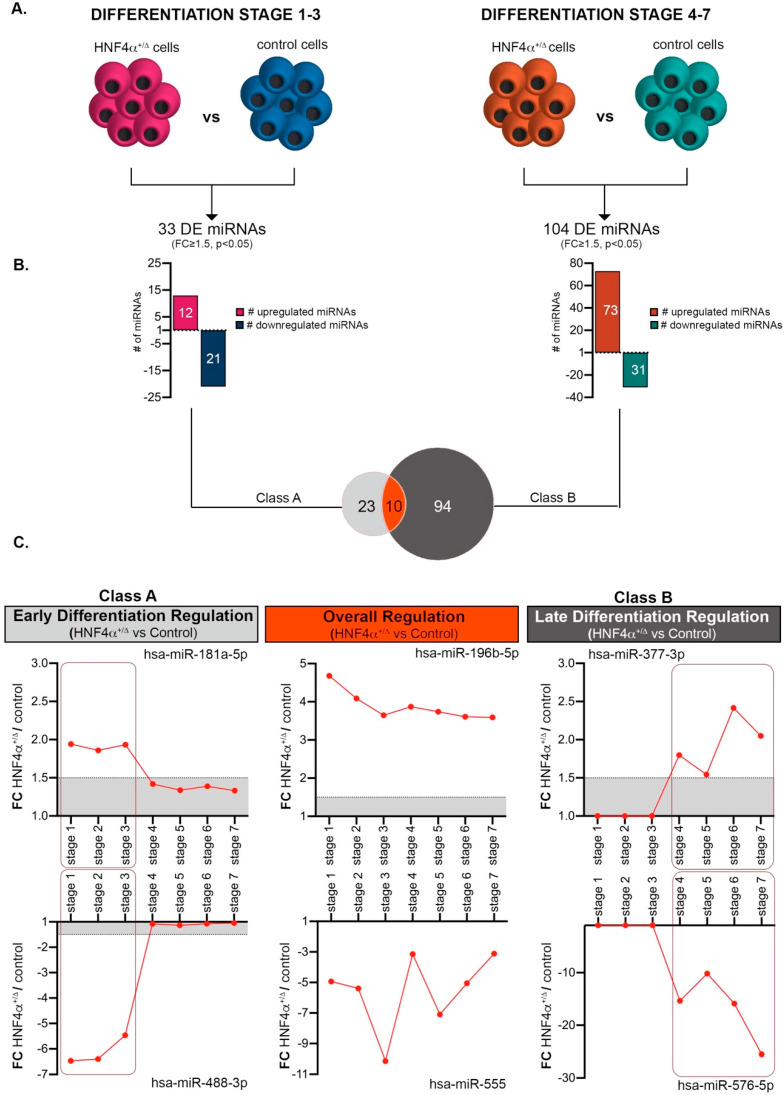
Comparative analysis of HNF4α+/Δ and control miRNAome during the early and late differentiation windows. (**A**) Experimental design of the condition pairs compared. (**B**) Number of miRNAs differentially expressed between HNF4α+/Δ and control cells during either early or late differentiation. (**C**) Selected representative RNAs’ regulation patterns (*n* = 1–3 samples per stage for controls; *n* = 2–6 samples per stage for HNF4α^+/Δ^). Boxed areas are showing the regulation switch after stages 1-3 for early differentiation effects (left boxes) and stages 4-6 for late differentiation effects (right boxes). Graphs’ data are given as mean ± SD.

To focus on the miRNA landscape changes caused by the HNF4α^+/Δ^ mutation, we subsequently selected miRNAs exhibiting significant global differences between HNF4α^+/Δ^ (MODY1) patients and their corresponding healthy controls during: (1) early (Class A, Table S1) or (2) late stages of differentiation (Class B, Table S1) (Figure 3A). We considered only the miRNAs having a normalized level of expression of at least 1 in at least one condition of the comparison. Class A consisted of 33 differentially expressed miRNAs (DEmiRs) between HNF4α^+/Δ^ and control-derived cells (Figure 3B,C). Almost two thirds of these (63.63% 21/33) were downregulated in the mutation carrier cells (i.e., presenting higher expression in the control-derived samples). Of note, during late differentiation (Class B), there were three times more miRNAs deregulated as a consequence of HNF4α^+/Δ^ mutation (3.15x, 104 vs. 33) (Figure 3B,C). Moreover, in contrast with the previous condition, these were largely upregulated (70.19% 73/104), indicating that, at later stages during differentiation, the mutation is either inducing miRNA production or impedes mechanisms involved in their degradation (Figure 3B). Finally, ten miRNAs were significantly regulated between the HNF4α^+/Δ^ mutation and control during both periods (i.e., all along the differentiation timeline, Figure 3B,C).

### 3.4. During Early Differentiation, HNF4α^+/Δ^ Mutation Impacts miRNAs Involved in Cell Cycle Regulation and Consequently Pancreatic Endoderm Cell Fate Acquisition 

**Figure 4:**
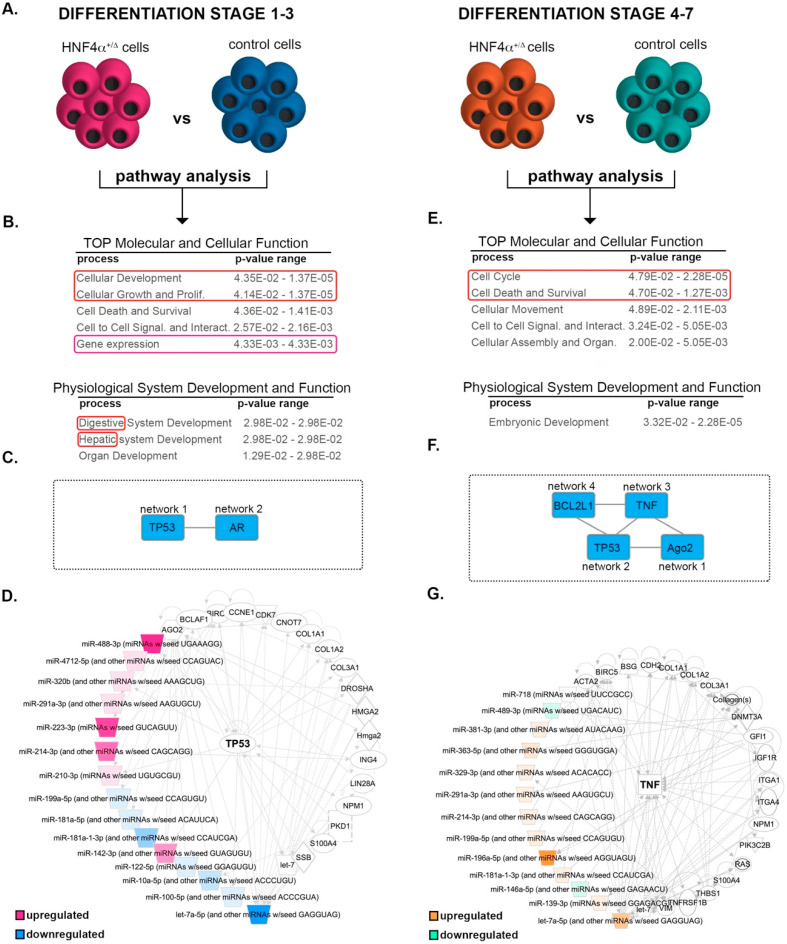
Pathway analysis of the differentially expressed miRNAs between the HNF4α^+/Δ^ mutation and control cells. (**A**) Experimental design of the condition pairs compared. (**B**) IPA-generated tables of the top molecular and cellular function (developmental cellular processes are marked by the red box, gene expression by purple box) and physiological system development and function for the differential miRNA landscape during the early differentiation period (red box marks the endocrine development signature). (**C**) IPA-generated overlapping networks. (**D**) IPA-generated network. (**E**) IPA-generated tables of the TOP molecular and cellular function and physiological system development and function for the differential miRNA landscape during the late differentiation period (proliferation /survival cellular processes are marked by the red box). (**F**) IPA-generated overlapping networks. (**G**) IPA-generated network.

Subsequently, to understand the putative function and the impact of the miRNA changes characterizing the HNF4α^+/**Δ**^, we explored the global DEmiRs landscapes of the two differentiation windows using the Ingenuity Pathway Analysis software (Figure 4A). As expected, the analysis revealed cellular development and cell growth and proliferation in the top molecular and cellular functions of the DEmiRs characterizing the early differentiation period (Class A, Figure 4B). Interestingly, it also identified digestive and hepatic system development as leading physiological functions. These results suggest that the HNF4α^+/Δ^ mutation affects the miRNAome early during differentiation and these differences already impact on the acquisition of the pancreatic endoderm cell fate. Furthermore, the network analysis of this miRNA set defined two distinct interconnected networks cantered on TP53 (Network 1)—a key cell cycle checkpoint regulator and AR (Androgen Receptor, Network 2)—a nuclear receptor involved in the regulation of cell cycle and senescence (Figure 4C and Supplemental Figure S1A). Interestingly, almost half of the deregulated miRNAs (45.45% 15/33) were networked around and under the regulation of TP53 (Figure 4D), implying their involvement in cell proliferation/differentiation. 

### 3.5. HNF4α^+/Δ^ Mutation Modulates miRNAs Involved in Cell Survival and Metabolic Processes during Late Differentiation 

The pathway analysis of the Class B miRNAs (i.e., differentially expressed between mutation carriers and controls during late differentiation) predicted cell cycle as well as cell death and survival as the top molecular and cellular functions (Figure 4E). Moreover, embryonic development was selected as the lead physiological function. In accordance, the network analysis defined several interconnected independent networks (Figure 4F) centered on Ago2 (Network 1, Supplemental Figure S1B), a protein involved in miRNA processing, TP53 (Network 2, Supplemental Figure S1C), TNF (Network 3, Figure 4G), an important hub for inflammation and metabolic processes, and BCL2L1 Network 4, Supplemental Figure S1D), a critical apoptosis regulator. These data indicate that the miRNA deregulated at later differentiation points as a result of the HNF4α^+/Δ^ mutation are involved in a broader range of processes as compared to their Class A counterparts. Moreover, the common centering on TP53 (network 1 Class A and network 2 Class B) indicates the involvement of miRNAs regulated during both differentiation windows. 

### 3.6. Transcriptome–miRNAome Analysis Revealed that miRNAs Deregulated in HNF4α^+/Δ^ Cells during Early Differentiation Cooperatively Target Critical Regulatory Hubs of Differentiation 

**Figure 5:**
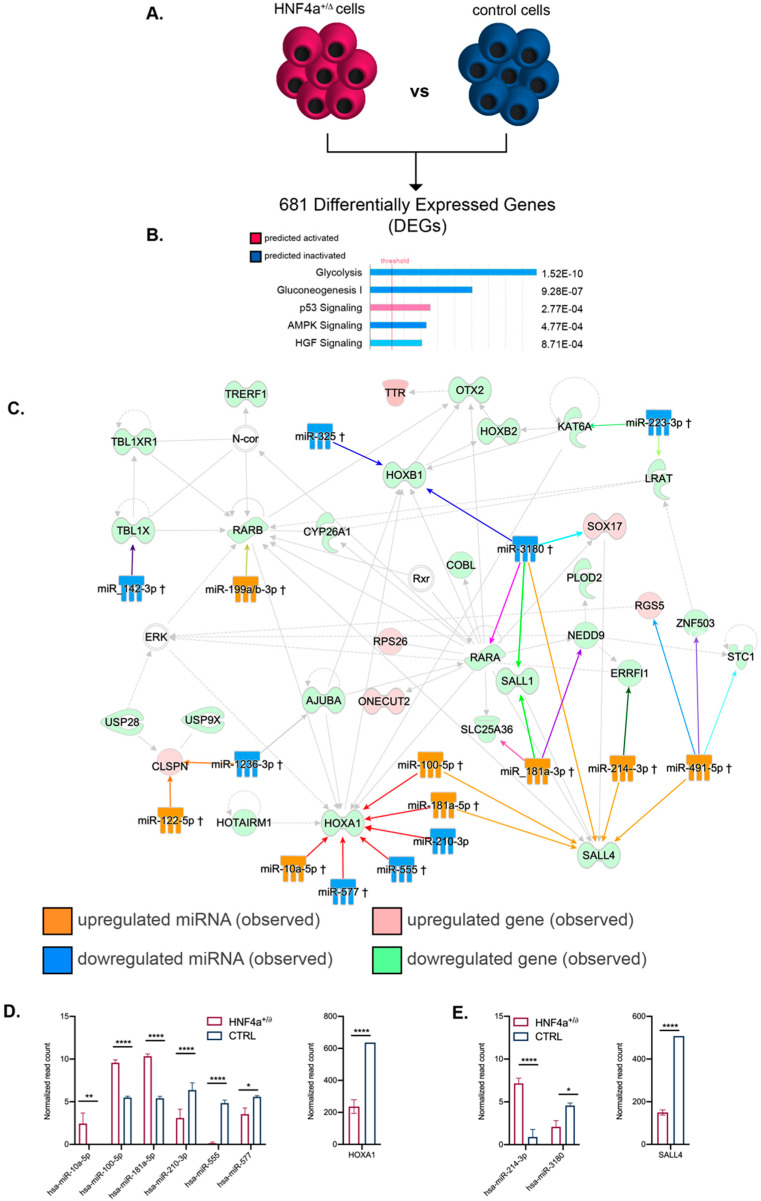
Pathway analysis of the mRNA–miRNA landscapes during the early differentiation period. (**A**) Experimental design. (**B**) IPA-generated top canonical pathways (absolute value z-score > 1) (**C**) miRNA–mRNA interactions of the top transcriptional landscape network. (**D**) Expression levels of miRNAs targeting the HOXA1 node and its respective expression level at stage 3 (*n* = 3 samples for controls; *n* = 6 samples for HNF4α^+/Δ^). (**E**) Expression levels of hsa-miR-214-3p and hsa-miR-3180 and their target SALL4 expression at stage 3 (*n* = 3 samples for controls; *n* = 6 samples for HNF4α^+/Δ^). Graphs data are shown as mean and SD. * *p* < 0.05, ** *p* < 0.01, **** *p* < 0.0001 (Mann–Whitney test).

**Figure 6:**
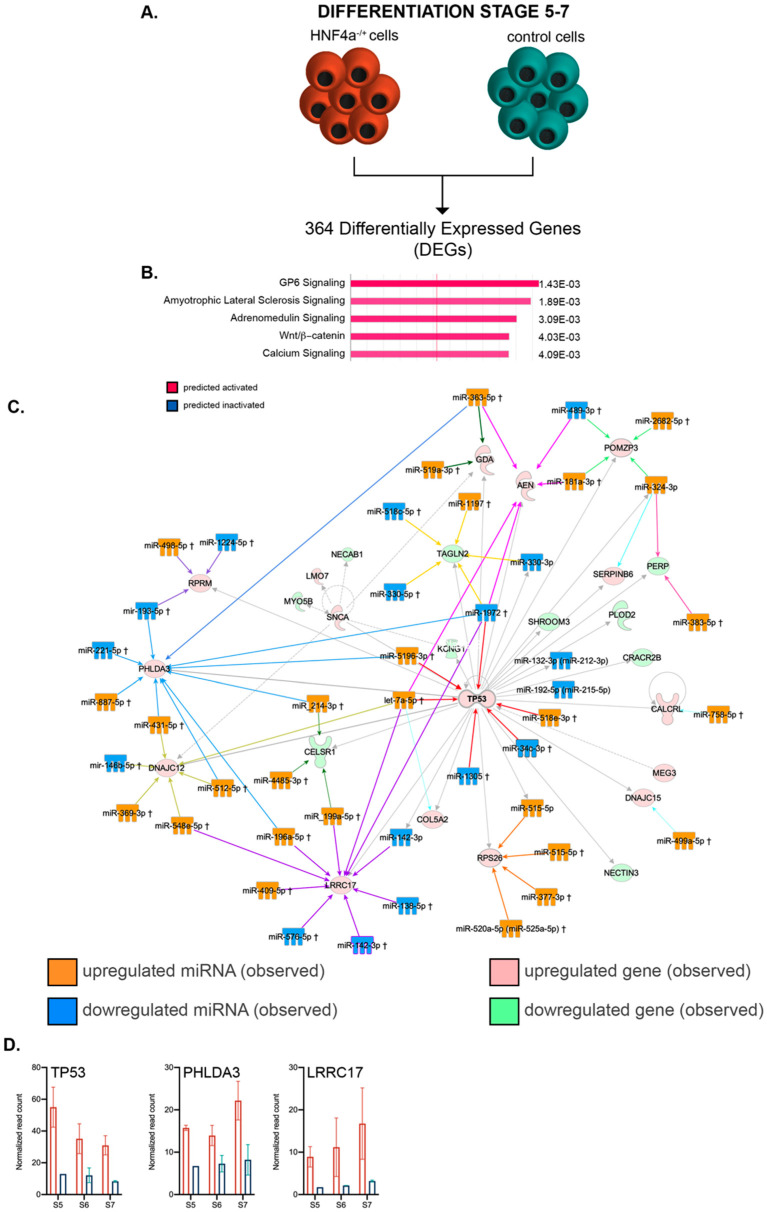
Pathway analysis of the mRNA–miRNA landscapes during the late differentiation period. (**A**) Experimental design. (**B**) IPA-generated top canonical pathways (absolute value z-score greater than 1). (**C**) miRNA–mRNA interactions of the top transcriptional landscape network. (**D**) Transcript expression levels of the interaction nodes: TP53, PHLDA3 and LRRC17 at stages 5, 6 and 7 in HNF4α^+/Δ^ (light orange) and control (green) lines (*n* = 2–6 samples per time-point).

To expand our analysis of the processes and cellular networks affected by the changes induced by HNF4α^+/Δ^ mutation in the miRNA landscape, we combined our previous analysis with a parallel global transcriptomics assay performed on the exact same samples (Figure 5 and Figure 6). Globally, 681 genes were differentially expressed (DEGs) between HNF4α^+/Δ^ mutation and controls during the early period of differentiation (Figure 5A). The pathway analysis revealed that these genes were involved in glucose metabolism, p53 signaling and protein biosynthesis (Figure 5B). Interestingly, the program inferred the inactivation of glycolysis, gluconeogenesis, and AMPK signaling, suggestive of low protein biosynthesis. In contrast, p53 signaling was predicted as activated, indicative of increased cell cycle arrest of the HNF4α^+/Δ^ cells and potentially apoptosis. This observation was further confirmed by TP53 being predicted as the top upstream regulator responsible for the observed regulatory landscape. Moreover, MYC, a potent transcriptional regulator of cell cycle genes, was predicted to be inactivated.

Besides being predicted as the leading transcription regulator (position 3, Table 2), SOX2 was also observed to be downregulated (−3.801x) in our assay, suggesting that HNF4α^+/Δ^ cells are more prone to exit pluripotency and engage in differentiation. 

We subsequently used IPA and combined the miRNA and their corresponding gene targets differentially expressed between HNF4α^+/Δ^ and control samples during the early differentiation window. Due to the large amount of data generated, for this study we focused on the top regulatory network characterizing the analyzed landscape (Figure 5C).

The analysis indicated two convergence nodes centered on homeotic genes, each of these being regulated by six distinct DEmiRs (two in common). The first group of miRNAs, centered on HOXA1 (converging red arrows on the interactome), exhibited mixed regulation (three upregulated, three downregulated). Nevertheless, the resulting observed downregulation of HOXA1 transcription suggests an active repression result (Figure 5D), implying their role in regulating retinoic acid (RA)-induced differentiation.

The second group of DEmiRs are centered on SALL4 (Sal-like protein 4, converging orange arrows). This is a transcription factor with a known critical role in stemness maintenance [30,31,32]. All but one (5/6) of the miRNAs targeting this node are observed upregulated in the HNF4α^+/Δ^ as compared to the control samples. This correlates with the observed downregulation of SALL4 gene expression (Figure 5E). In contrast, other transcriptional regulators, such as OTX2 or HOXB1, were targeted by just a single DEmiR of the dataset. Furthermore, several DEmiRs targeted multiple distinct nodes, such as miR-3180 (observed downregulated) and mir-491-5p (observed upregulated) with six and five gene targets, respectively. 

Globally, these data suggest that the HNF4α^+/Δ^ cells differentiate earlier than their control counterparts; nevertheless, a wider study of the top networks and gene candidates should be performed to further confirm this observation.

### 3.7. The mRNA-miRNA Interactome of HNF4α^+/Δ^ Cells during the Last Stages of Differentiation Confirms the Involvement of DEmiRs in Cell Cycle Regulation Arrest

Similar to the above global transcriptome analysis during early differentiation, we addressed the transcriptional landscape characterizing the HNF4α^+/Δ^ cells during late differentiation (Figure 6A). The pathway analysis of the 364 DEGs between HNF4α^+/Δ^ and control cells (FC ≥ 2, *p* < 0.05) inferred that pathways involved in transducing cellular stress (such as GP6 and the Amyotrophic Lateral Sclerosis Pathway), cell survival (such as the Adrenomedullin Signaling Pathway, the activation of which is linked to growth stimulation and apoptosis inhibition) and differentiation (such as Wnt/β–catenin signaling) were all predicted to be activated based on the observed transcriptome landscape (Figure 6B). In agreement, critical transcription factors for differentiation and development, such as SOX2, β-catenin (CTNNB1) and EOMES, were selected as top upstream regulators (Table 3), with β-catenin being the top predicted activated molecule of the assay (activation z-score 3.235). 

Of note, the program pinpointed HNF4α inhibition as a top regulator responsible for the observed landscape (Table 4). 

As before, by using IPA, we focused our analysis on the leading regulatory network characterizing the global transcriptional signature of HNF4α^+/Δ^ cells and matched the DEmiRs to their corresponding networked DEGs (Figure 6C). Of interest, the main network centered on TP53, which was observed to be upregulated in the assay (Figure 6D), once more indicating increased cell cycle arrest in the HNF4α^+/Δ^ samples. 

Although the capping value for network size was the same for both early and late differentiation analyses (35 molecules), the interactome of the latter revealed multiple convergence nodes, defined by targets aimed at by at least four different DEmiRs (8 as compared to 2). As such, PHLDA3 (observed upregulated, blue converging arrows), a p53-regulated AKT signaling repressor with a role in apoptosis induction, is targeted by as many as 10 distinct DEmiRs (Figure 6C). Interestingly, the upregulation of this gene was also shown to be critical for blocking the growth of pancreatic neuroendocrine tumors via AKT inhibition [33].

A second node targeted by 10 different DEmiRs was centered on LRRC17 (observed upregulated, purple converging arrows, Figure 6C), with only one miRNA shared with the previous PHLDA3-centered set. LRRC17 was previously involved in the inhibition of differentiation in other systems [34,35]. 

The central node of the network, TP53, was targeted by six distinct DEmiRs (red converging arrow). Interestingly, all the three nodes described above were targeted by an equal number of upregulated and downregulated DEmiRs, suggesting additional levels of complexity modulating the target molecules.

Overall, these data suggest that HNF4α^+/Δ^ cells further undergo p53-regulated cell cycle arrest, evolving from predicted regulation during the early differentiation window to experimentally confirmed observations during the late stages. 

## 4. Discussion

To the best of our knowledge, this is the first differentiation-wide investigation addressing the miRNAome of MODY1 patient-derived islet-like cells and thus the impact of HNF4α^+/Δ^ mutation at post-transcriptional levels. Previous studies involving MODY1 patients bearing the same mutation did not cover miRNA regulation [21,22]. However, one study [22] also pinpointed affected foregut endoderm gene expression signatures in the HNF4α^+/Δ^ cells, although it employed a different differentiation protocol. We also previously showed that the heterozygous state of HNF4α mutation does not interfere with the acquisition of the β-cell fate in hiPSC-derived islet-like cells (S6 and S7) by using global proteomics [21]. Moreover, HNF4α plays a crucial role in vivo in restricting the pancreatic endocrine cell fate towards a monohormonal choice [23]. 

In this study, we compared the miRNAome of differentiating hiPSC cells derived from HNF4α^+/Δ^ mutation carriers (MODY1) and their family control along the differentiation timeline. The analysis of miRNA modulations highlighted a steep difference in the regulation pattern occurring during the transition from the posterior gut to pancreatic endoderm, defining an early and a late differentiation regulatory window. The number of miRNAs differentially expressed between HNF4α^+/Δ^ cells and their corresponding controls increased from early to late differentiation, suggesting a more important impact of the mutation following the acquisition of the pancreatic progenitor fate. The pathway analysis of the miRNAome, transcriptome and miRNA–mRNA interactome revealed a likely gradual involvement of HNF4α^+/Δ^ mutation in p53-mediated cell cycle arrest, with consequences for the proliferation potential, survival and cell fate acquisition of the differentiating cells. Judging by the miRNA interaction partners’ regulation, the cell cycle arrest seems to promote premature differentiation during early differentiation; however, this needs to be formally proved by future studies.

It should be stated that, due to the numerous miRNAs having potential to regulate the same target [36], clear conclusions about each miRNA’s function and its transcript partner are difficult to reach. Moreover, many of the miRNA transcript partners are based on theoretical inference and were never demonstrated experimentally, so it is entirely possible that some DEmiR–DEG interactions are only possible at a theoretical level. In addition, it should be considered that the effects of HNF4α^+/Δ^ mutations on the miRNAome profile could be direct, but also indirect, through the many HNF4α target genes. Even more, some of the miRNA regulations observed in this study could arise as a compensatory response to HNF4α^+/Δ^-based changes in gene expression. Nevertheless, our study clearly indicates that there is no evident master miRNA involved in regulating gene expression in response to HNF4α^+/Δ^ mutation, but a larger subset of critical, possibly redundant, miRNAs. Consequently, validating the miRNA–target gene interactions in this context will require tedious work involving demultiplexing the collective effects of potentially redundant miRNAs, exhibiting different regulations and colliding on the same target. Thus, any future attempts for “rescuing” the regulation should probably be focused at multiple miRNA targets. Moreover, it should be considered that tampering with the p53 machinery and thus cell cycle control components will heavily impact on cell molecular signature and function, with consequences far beyond HNF4α-mutation-induced changes in the miRNA signature. Additionally, due to the high redundancy of the cell cycle control mechanisms, the deregulation of more than one miRNA will probably be required, increasing the difficulty of demultiplexing the observed changes and especially of reliably connecting them to the HNF4α-induced change in the pattern of a certain miRNA.

The in-vitro character of the approach represents another inherent drawback of this study, as the observed landscape characterizes cells outside of their normal niche and in the absence of the typical in-vivo interactions. Moreover, the current experimental setup does not allow discriminating between the different cell subpopulations generated along the differentiation line. Hence, the observed regulations represent the resultant heterogeneous cell population. It should also be considered that our analysis included only one HNF4α family—thus, one control and two affected individuals—due to the limited number of available samples. Moreover, the genetic background of siblings can differ significantly and, in order to reduce noise, it would be preferable to use isogenic controls generated using genome-editing tools (i.e., CRISPR-Cas9 technology) rather than family controls in any future study. Nevertheless, we expect that valuable information can be inferred from the present study, limiting the number of potential miRNA targets to be experimentally validated in a future study focused on knockdown/knockout of selected miRNA in transgenic animal models, in order to further elucidate the disease mechanism in MODY1. We expect that, pending experimental validation, certain miRNAs deregulated in the HNF4α^+/Δ^ cells involved in cell cycle arrest on multiple targets, such as miR-3180, miR-1972 and let-7a-5p, could prove useful for therapy or in preventing the onset of MODY1. Moreover, the miRNA signature of healthy cells presented here could also provide a good reference for other studies focusing on different aspects of endocrine differentiation. 

## Figures and Tables

**Table 1:** Donor patients and healthy sibling details.

Name	Age	Gender	Family Relation	Mutation
Donor 1	51	male	father	Heterozygous *p.Ile271fs*
Donor 2	26	male	son	Heterozygous *p.Ile271fs*
Donor 3	31	male	son	none

**Table 2:** Top upstream regulators predicted by IPA acting in early differentiation stages.

Name	*p*-Value	Details
TP53	6.82 × 10^−25^	Transcription regulator
HIF1A	2.62 × 10^−24^	Transcription regulator
SOX2	9.15 × 10^−23^	Transcription regulator
EGLN	7.76 × 10^−22^	(group)
MYC ^1^	6.25 × 10^−19^	(−3.604 activation z- score)

^1^ inferred to be inactivated by IPA.

**Table 3:** Top upstream regulators predicted by IPA acting during late differentiation stages.

Name	*p*-Value	Molecule Type
SOX2	5.86 × 10^−12^	Transcription regulator
CTNNB1	1.70 × 10^−10^	Transcription regulator
HNRNPA2B1	1.61 × 10^−9^	
EOMES	1.22 × 10^−8^	Transcription regulator
TAF4	3.51 × 10^−8^	

**Table 4:** Top upstream transcription regulators predicted by IPA to be inhibited in during late differentiation.

Name	Molecule Type	*p*-Value	Activation Z-Score
EOMES	Transcription regulator	1.22 × 10^−8^	−2.496
REST	Transcription regulator	4.33 × 10^−4^	−2.189
HNF4α	Transcription regulator	7.46 × 10^−2^	−2.000

## Supplementary Materials

The following are available online at https://www.mdpi.com/2227-9059/8/7/179/s1, Figure S1: IPA-generated networks of the differentially expressed miRNAs. Table S1: differentially expressed miRNAs (DEmiRs).
