# Supplementary material for: Bioinformatic Analyses of miRNA–mRNA Signature during hiPSC Differentiation towards Insulin-Producing Cells upon HNF4α Mutation
Source: Biomedicines. 2020 Jun 27;8(7):179. doi: 10.3390/biomedicines8070179 (PMC7400504; doi:10.3390/biomedicines8070179)
Supplement: Supplementary file 1 [file biomedicines-08-00179-s001.pdf]

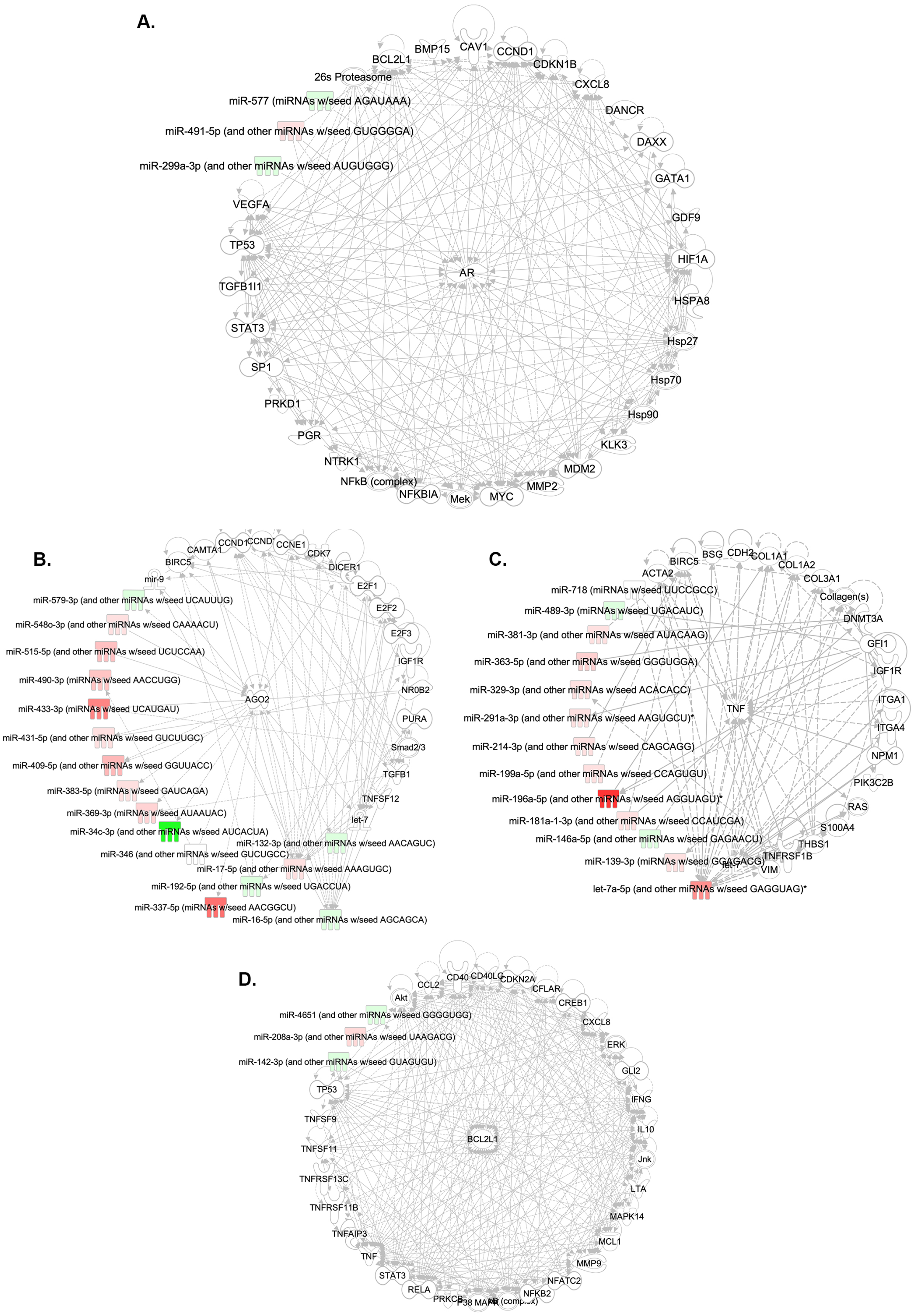

Supplemental Figure 1: A) IPA generated network of the differentially expressed miRNAs between the HNF4$^{\alpha+/\Delta}$ mutation and control cells during early differentiation. **B-D)** IPA generated networks of the differentially expressed miRNAs between the HNF4$^{\alpha+/\Delta}$ mutation and control cells during the late differentiation window.

Table S1: List of differentially expressed miRNAs (DEmiRs):

CONTROL CELLS (EARLY vs LATE DIFFERENTIATION)	HNF4α+/Δ CELLS (EARLY vs LATE DIFFERENTIATION)	EARLY DIFFERENTIATION (HNF4α+/Δ vs CONTROL)	LATE DIFFERENTIATION (HNF4α+/Δ vs CONTROL)
hsa-miR-1261	hsa-miR-1281	hsa-miR-488-3p	hsa-miR-34c-3p
hsa-miR-761	hsa-miR-608	hsa-miR-223-3p	hsa-miR-576-5p
hsa-let-7d-5p	hsa-miR-138-5p	hsa-miR-761	hsa-miR-664b-3p
hsa-miR-519d-3p	hsa-miR-1257	hsa-miR-142-3p	hsa-miR-325
hsa-miR-595	hsa-miR-489-3p	hsa-miR-555	hsa-miR-758-5p
hsa-miR-770-5p	hsa-miR-215-5p	hsa-miR-520c-3p	hsa-miR-195-5p
hsa-miR-517c-3p+hsa-miR-519a-3p	hsa-miR-1972	hsa-miR-521	hsa-miR-518b
hsa-miR-572	hsa-let-7d-5p	hsa-miR-1261	hsa-miR-146b-5p
hsa-miR-1307-5p	hsa-miR-212-3p	hsa-miR-770-5p	hsa-miR-142-3p
hsa-miR-520a-5p	hsa-miR-548ah-5p	hsa-miR-1236-3p	hsa-miR-941
hsa-miR-373-3p	hsa-miR-498	hsa-miR-514a-3p	hsa-miR-181b-2-3p
hsa-miR-223-3p	hsa-miR-512-5p	hsa-miR-1307-5p	hsa-miR-555
hsa-miR-4485-3p	hsa-miR-302a-5p	hsa-miR-595	hsa-miR-221-5p
hsa-miR-1236-3p	hsa-miR-373-3p	hsa-miR-572	hsa-miR-608
hsa-miR-514a-3p	hsa-miR-1307-3p	hsa-miR-325	hsa-miR-499a-5p
hsa-miR-520c-3p	hsa-miR-1323	hsa-miR-3180	hsa-miR-548d-5p
hsa-miR-3180	hsa-miR-488-3p	hsa-miR-210-3p	hsa-miR-138-5p
hsa-miR-1257	hsa-miR-329-3p	hsa-miR-299-3p	hsa-miR-1224-5p
hsa-miR-142-3p	hsa-miR-383-5p	hsa-miR-320a	hsa-miR-105-5p
hsa-miR-1281	hsa-miR-889-3p	hsa-miR-651-5p	hsa-miR-489-3p
hsa-miR-608	hsa-miR-760	hsa-miR-577	hsa-miR-330-3p
hsa-miR-138-5p	hsa-miR-301b-5p	hsa-miR-122-5p	hsa-miR-215-5p
hsa-miR-325	hsa-miR-542-3p	hsa-miR-100-5p	hsa-miR-760
hsa-miR-372-3p	hsa-miR-941	hsa-miR-181a-5p	hsa-miR-1972
hsa-miR-521	hsa-miR-887-5p	hsa-miR-199a-3p+hsa-miR-199b-3p	hsa-miR-34c-5p
hsa-miR-215-5p	hsa-miR-654-5p	hsa-miR-199a-5p	hsa-miR-548d-3p
hsa-miR-489-3p	hsa-miR-654-3p	hsa-miR-10b-5p	hsa-miR-193a-5p+hsa-miR-193b-5p
hsa-miR-1972	hsa-miR-1185-1-3p	hsa-miR-214-3p	hsa-miR-330-5p
hsa-miR-212-3p	hsa-miR-2682-5p	hsa-miR-491-5p	hsa-miR-548ah-5p
hsa-miR-548ah-5p	hsa-miR-615-3p	hsa-miR-10a-5p	hsa-miR-1305
hsa-miR-1298-5p	hsa-miR-490-3p	hsa-miR-181a-3p	hsa-miR-212-3p
hsa-let-7g-5p	hsa-miR-4707-5p	hsa-let-7g-5p	hsa-miR-521
hsa-let-7c-5p	hsa-miR-409-5p	hsa-let-7d-5p	hsa-let-7i-5p
hsa-miR-485-3p	hsa-miR-377-3p	hsa-let-7c-5p	hsa-miR-199a-5p
hsa-miR-299-5p	hsa-miR-499a-5p		hsa-let-7b-5p
hsa-let-7f-5p	hsa-miR-221-5p		hsa-miR-378g
hsa-miR-105-5p	hsa-miR-1224-5p		hsa-miR-1323
hsa-miR-664b-3p	hsa-miR-330-3p		hsa-let-7a-5p
hsa-miR-576-5p	hsa-miR-146b-5p		hsa-miR-206
hsa-miR-324-3p	hsa-miR-30a-5p		hsa-miR-372-3p
hsa-miR-10a-5p	hsa-miR-345-5p		hsa-let-7f-5p

hsa-miR-491-5p	hsa-miR-1260b	hsa-miR-496
hsa-miR-206	hsa-miR-210-3p	hsa-let-7g-5p
hsa-miR-496	hsa-miR-378g	hsa-let-7c-5p
hsa-miR-499a-5p	hsa-miR-200a-3p	hsa-miR-324-3p
hsa-miR-221-5p	hsa-miR-95-3p	hsa-miR-4531
hsa-miR-1224-5p	hsa-miR-362-3p	hsa-miR-383-5p
hsa-miR-330-3p	hsa-miR-483-3p	hsa-miR-887-5p
hsa-miR-146b-5p	hsa-miR-141-3p	hsa-miR-548y
hsa-miR-95-3p	hsa-miR-10b-5p	hsa-miR-520a-5p
hsa-miR-744-5p	hsa-miR-10a-5p	hsa-miR-432-5p
hsa-miR-10b-5p	hsa-miR-521	hsa-miR-4485-3p
	hsa-miR-206	hsa-miR-498
	hsa-miR-29c-3p	hsa-miR-373-3p
	hsa-miR-381-3p	hsa-miR-196a-5p
	hsa-miR-429	hsa-miR-370-3p
	hsa-miR-555	hsa-miR-514b-5p
	hsa-miR-330-5p	hsa-miR-1197
	hsa-miR-496	hsa-miR-139-3p
	hsa-miR-190a-5p	hsa-miR-98-5p
	hsa-miR-337-5p	hsa-miR-381-3p
	hsa-miR-491-5p	hsa-miR-214-3p
	hsa-miR-433-3p	hsa-miR-431-5p
		hsa-miR-1253
		hsa-miR-519d-3p
		hsa-miR-889-3p
		hsa-miR-181a-3p
		hsa-miR-888-5p
		hsa-miR-526a+hsa-miR-518c-5p+hsa-miR-518d-5p
		hsa-miR-517c-3p+hsa-miR-519a-3p
		hsa-miR-548e-5p
		hsa-miR-891b
		hsa-miR-654-5p
		hsa-miR-512-5p
		hsa-miR-329-3p
		hsa-miR-654-3p
		hsa-miR-1185-1-3p
		hsa-miR-4707-5p
		hsa-miR-563
		hsa-miR-585-3p
		hsa-miR-495-5p
		hsa-miR-1185-2-3p
		hsa-miR-615-3p
		hsa-miR-2682-5p
		hsa-miR-518e-3p
		hsa-miR-513a-3p

hsa-miR-208b-3p
hsa-miR-301b-5p
hsa-miR-369-3p
hsa-miR-4536-5p
hsa-miR-363-5p
hsa-miR-490-3p
hsa-miR-515-5p
hsa-miR-5196-3p+hsa-miR-6732-3p
hsa-miR-409-5p
hsa-miR-525-5p
hsa-miR-526b-5p
hsa-let-7d-5p
hsa-miR-377-3p
hsa-miR-512-3p
hsa-miR-433-3p
hsa-miR-337-5p
hsa-miR-181a-2-3p
hsa-miR-196b-5p
